# Median Nerve Axonotmesis Following Bleomycin Electrosclerotherapy: A Case Study of a Significant Complication After Treatment of a Lymphovascular Malformation

**DOI:** 10.7759/cureus.100739

**Published:** 2026-01-04

**Authors:** Graeme M Downes, Maria-Ioanna Gavala, Graham Collin, Ben Rymer

**Affiliations:** 1 Plastic Surgery, Royal Devon and Exeter Hospital, Exeter, GBR; 2 Interventional Radiology, North Bristol National Health Service (NHS) Trust, Bristol, GBR

**Keywords:** arm compartment syndrome, capillary-lymphatic-venous malformation, electro surgery, intralesional bleomycin sclerotherapy, median nerve injury

## Abstract

Bleomycin electrosclerotherapy (BEST) is a recently introduced treatment for lymphovascular malformations that combines intralesional bleomycin injection under image guidance with a precisely modulated electrical field to enhance cellular uptake. This technique is reported to have a favourable safety profile, with most adverse effects limited to transient pain and pigmentation. We report the case of a 20-year-old female patient who presented with severe, intractable pain in the left forearm and hand following BEST treatment for a forearm lymphangioma. Clinical examination demonstrated neuropathic pain with associated sensory deficit in the median nerve distribution. Due to concern for compartment syndrome, the surgical team performed urgent exploration of the forearm and a four-compartment fasciotomy. Postoperatively, the fasciotomy did not relieve symptoms, and subsequent clinical findings supported a diagnosis of direct, nonthermal electrical injury to the median nerve. Review of intraprocedural imaging revealed distortion of the electrode probe tip geometry. We hypothesize that this deformation resulted in an unintended increase in the focal electrical field, exceeding the threshold for irreversible electroporation, causing axonal injury, rather than the intended reversible electroporation required for drug delivery.

To our knowledge, this report describes the first documented case of median nerve axonotmesis following BEST. Deformation of the electrode geometry may significantly increase focal electrical field strength and result in unintended nerve injury. Although BEST demonstrates efficacy and an overall favourable safety profile in the treatment of lymphovascular malformations, clinicians should remain aware of this potential complication to support informed consent and appropriate management of postoperative presentations that may mimic compartment syndrome.

## Introduction

Bleomycin electrosclerotherapy (BEST) is a novel treatment modality first described for vascular malformations in 2017 by McMorrow and colleagues [1]. The technique involves direct infiltration of bleomycin into the malformation, followed by the application of a pulsed electric field to create discontinuity of the cell surface membrane with reversible electroporation, allowing for increased permeation of bleomycin to act as a sclerotherapy agent. This phenomenon was first observed in laboratory studies in 1988 [2]. BEST is generally regarded as safe, with commonly reported adverse effects limited to mild pain and skin pigmentation [3]. The combination of low-dose intralesional bleomycin with cliniporation allows effective treatment while reducing systemic exposure and the associated risk of pulmonary fibrosis [1]. Despite its increasing adoption, its neurovascular safety profile remains underreported.

We present a case in which a 20-year-old female patient attended her local emergency department in severe pain after BEST treatment for a lymphangioma of the left forearm. This presentation represents a significant complication of BEST treatment that appears to be previously undescribed. It is intended that highlighting this complication will enable better joint decision-making between clinicians carrying out BEST and their patients.

## Case presentation

**Figure 1:**
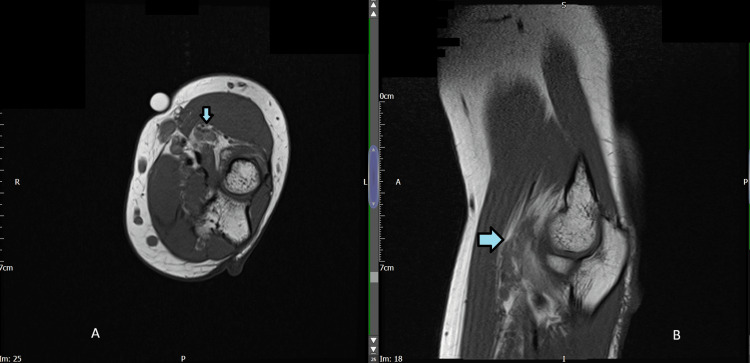
T1 Weighted MRI images of the patient T1-weighted MRI images at the level of the radial head with the lymphovascular malformation highlighted by arrows. Image A (axial) and image B (sagittal).

A 20-year-old female with a lifelong history of left forearm lymphangioma had been treated previously with debulking surgery at the age of two. She presented again with worsening discomfort and was investigated with an MRI, revealing an 8x3 cm serpiginous lymphangioma lying in the deep volar compartment (Figure 1). She was initially treated with intralesional bleomycin sclerotherapy, which failed to improve her symptoms. More detailed assessment of her symptoms confirmed that the majority of her discomfort was probably due to the bulk of the deep component within the supinator muscles, as she had pain on resisted supination and was unable to fully straighten her elbow.

Surgical debulking was considered and discounted, as the malformation was deep and diffuse, so that surgery would have been challenging; only if sclerosant options had been exhausted would surgery have been used. The potential for release of the posterior interosseous nerve (PIN) was also considered, but PIN impingement was felt less likely to be the primary cause of her symptoms.

She consented to bleomycin electrosclerotherapy with the stated aim of reducing the bulk and pain from her malformation and with the hope of improving arm function. Under general anaesthesia, 4,000 units of bleomycin were injected into the malformation under ultrasound and fluoroscopic control, and the area was treated using a total of three pulses of an Igea adjustable length hexagonal electrode (H-30-ST). A brief latent period followed; upon examination that evening, she had some tingling in her arm but no pain, and was discharged home as planned. Within hours of the procedure, she developed increasingly severe forearm and hand pain before presenting to the local emergency department the following morning. She was otherwise fit and well, took no regular medications, and was physically active, with a documented severe allergy to morphine.

**Figure 2:**
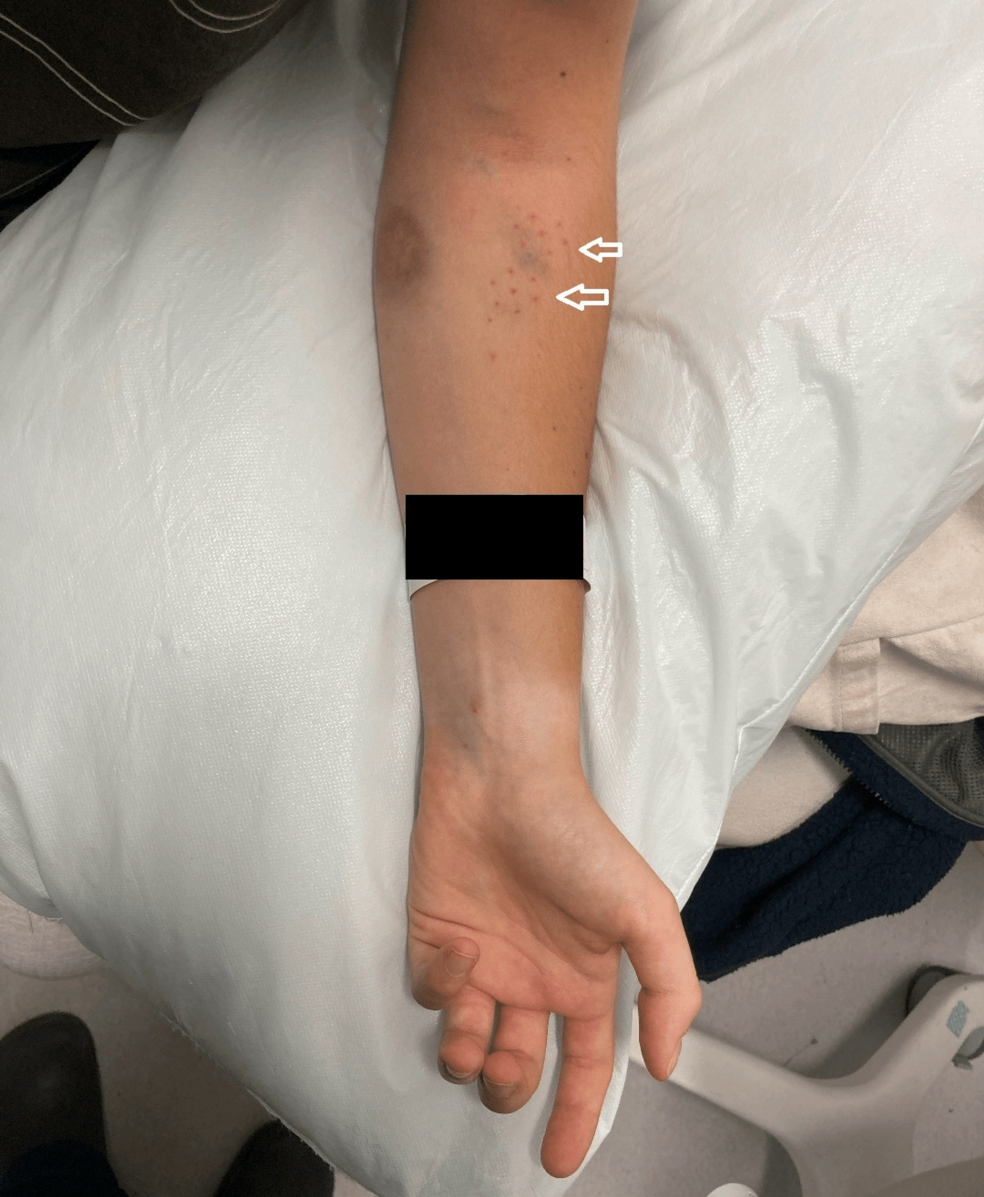
Initial presentation in the emergency department The sites of electrode entry can be seen in the proximal forearm, marked with arrows, as well as the abnormal posture of the fingers. The patient’s identity wristband has been redacted.

The initial assessment by the plastic surgery team revealed severe pain in the left forearm and hand with initial paraesthesia in the median nerve distribution, which progressed to global dysesthesia of the left upper limb. The patient was unable to actively move the hand, and any passive movements of the fingers were not tolerated. The hand was held with the thumb and index fingers extended (Figure 2) in a posture consistent with a hand of benediction sign, suggestive of a median nerve palsy. The forearm compartments were soft, but palpation was associated with extreme pain.

Surgical findings

Given the extreme, worsening pain and neurological deficit with associated possible inciting event (i.e., BEST), urgent decompression of the left forearm was arranged to treat clinically suspected compartment syndrome. This was carried out immediately after the assessment. When measured, the forearm compartment pressures were normal (volar 10-11 mmHg, dorsal 9 mmHg). The patient was placed under anaesthesia, and based on the clear clinical signs, a four-compartment fasciotomy was carried out. The compartments did not bulge or give a surgical impression of compartment syndrome upon release. Upon reflecting the brachioradialis, a tight, deep fascia around the lymphangioma and fibrotic tissue was found. No evidence of muscle ischaemia was present. The radial artery and vena comitans were reflected in an ulnar direction to expose the deep volar compartment, which also revealed tight fascia and epimysium, tissue fluid, and a contused superficial branch of the radial nerve (SBRN). The initial clinical impression was the development of a nonanatomical compartment around the tumour and proximal forearm, which increased in pressure following treatment, causing compression of the median nerve and SBRN in this neocompartment.

**Figure 3:**
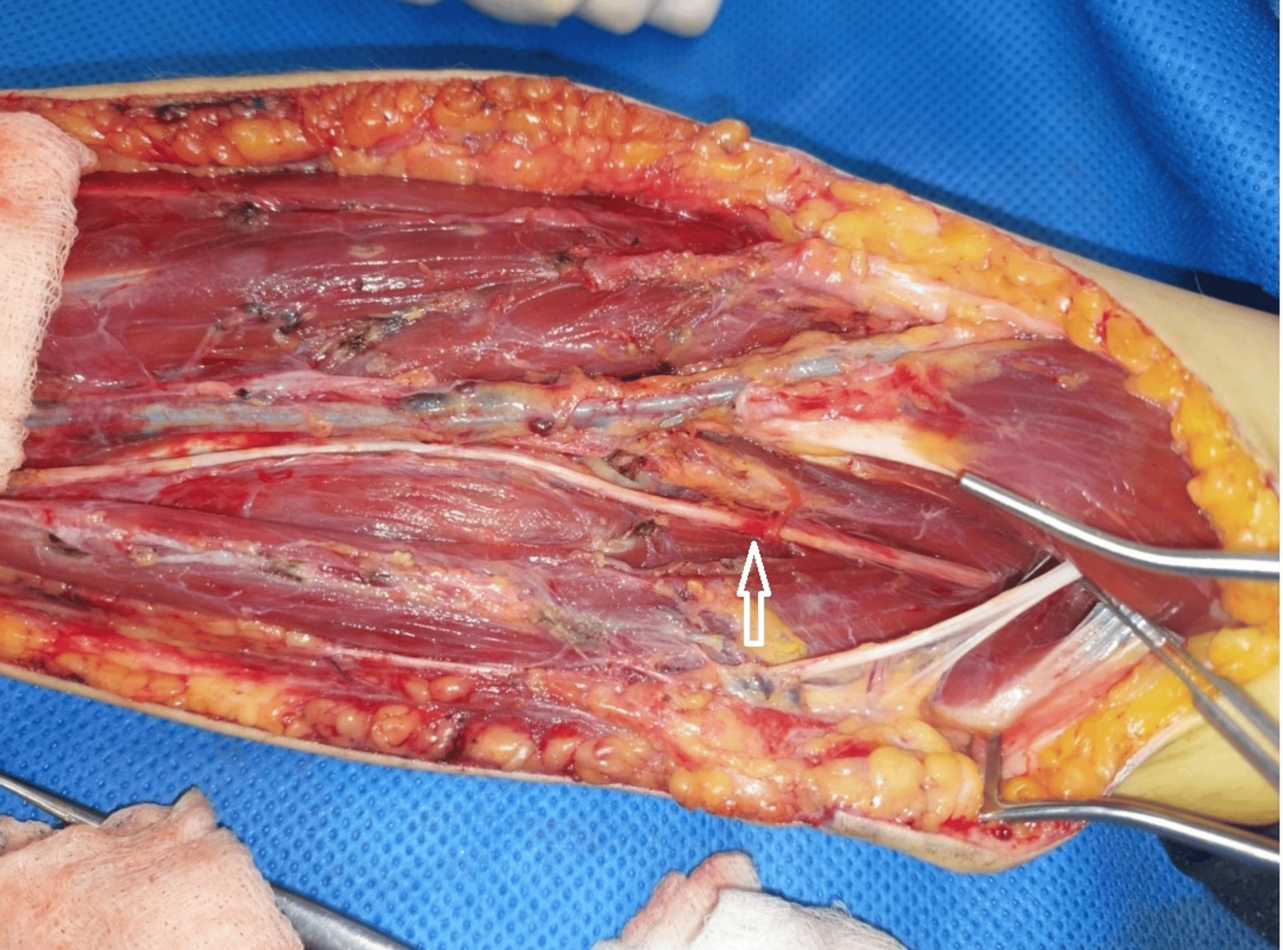
Intra-operative image Intra-operative appearances of the left forearm with the SBRN exposed centrally and marked with an arrow. The antecubital fossa is to the right of the image.

Upon regaining consciousness postoperatively, the patient was still in uncontrollable pain from her shoulder to her hand. Re-exploration for further external neurolysis and more proximal exploration were undertaken on the same day. During the second exploration, the radial, median, and lateral antebrachial cutaneous nerves were identified, fully explored, and released. Further thickened and scarred fibrous tissue covering the median nerve and SBRN was identified. Two points of obvious contusion on the SBRN and median nerve were identified (Figures 2, 3). These points were at the level of the antecubital fossa and corresponded exactly to where the tips of the BEST electrodes had sat, based on the skin entry points and confirmed length of the probes used. Inflammatory fluid in the distal biceps and a bruised flexor carpi radialis muscle, which was the deepest area of the BEST injection, were also noted. All nerves were released lengthwise. There was no macroscopic damage to the epineurium of any nerve explored.

Postoperative course

**Figure 4:**
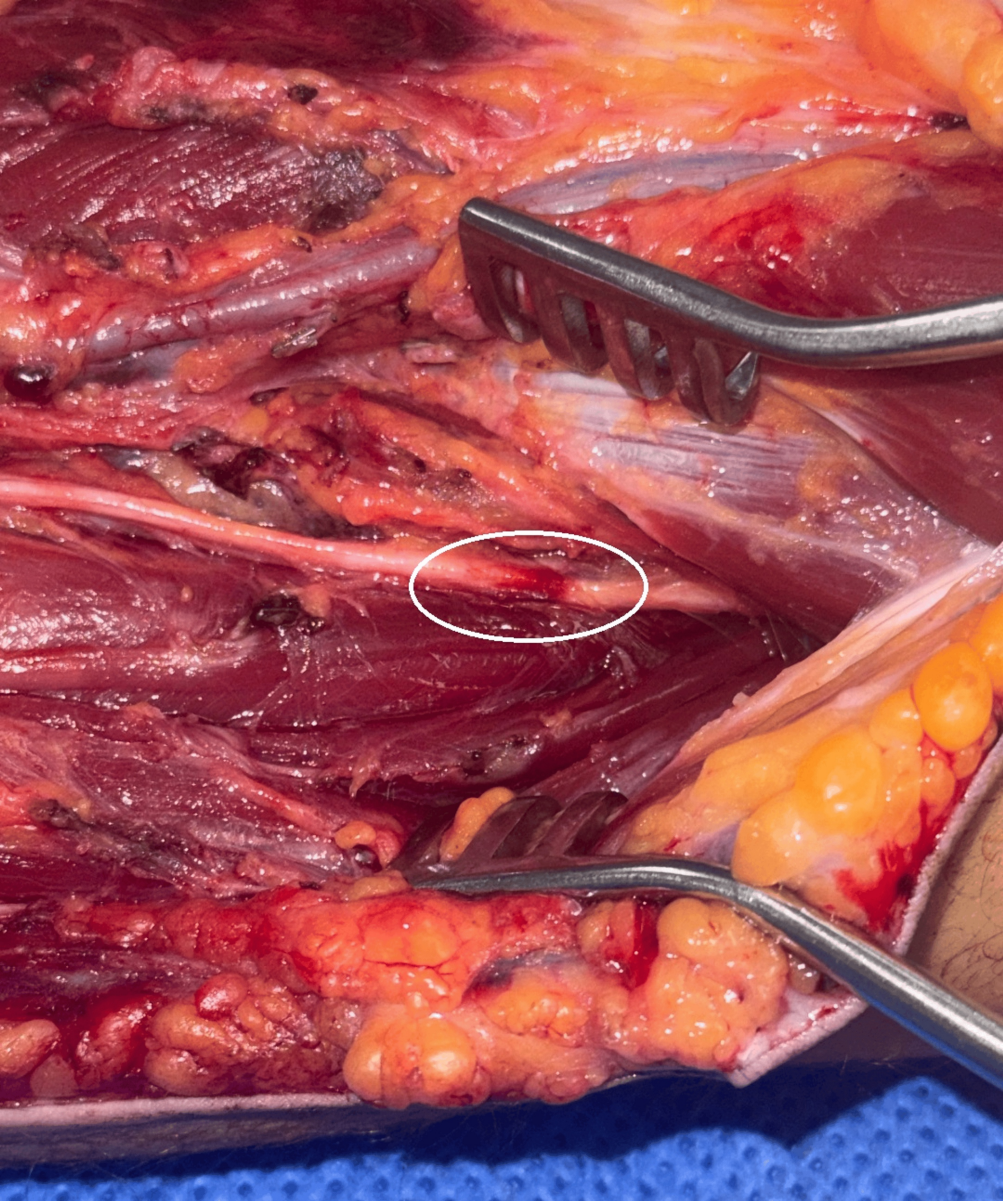
Intra-operative close-up image of the left forearm Intra-operative close-up view of the left forearm with the area of the superficial branch of the radial nerve (SBRN) contusion highlighted by a circle. The antecubital fossa is to the right of the image.

**Figure 5:**
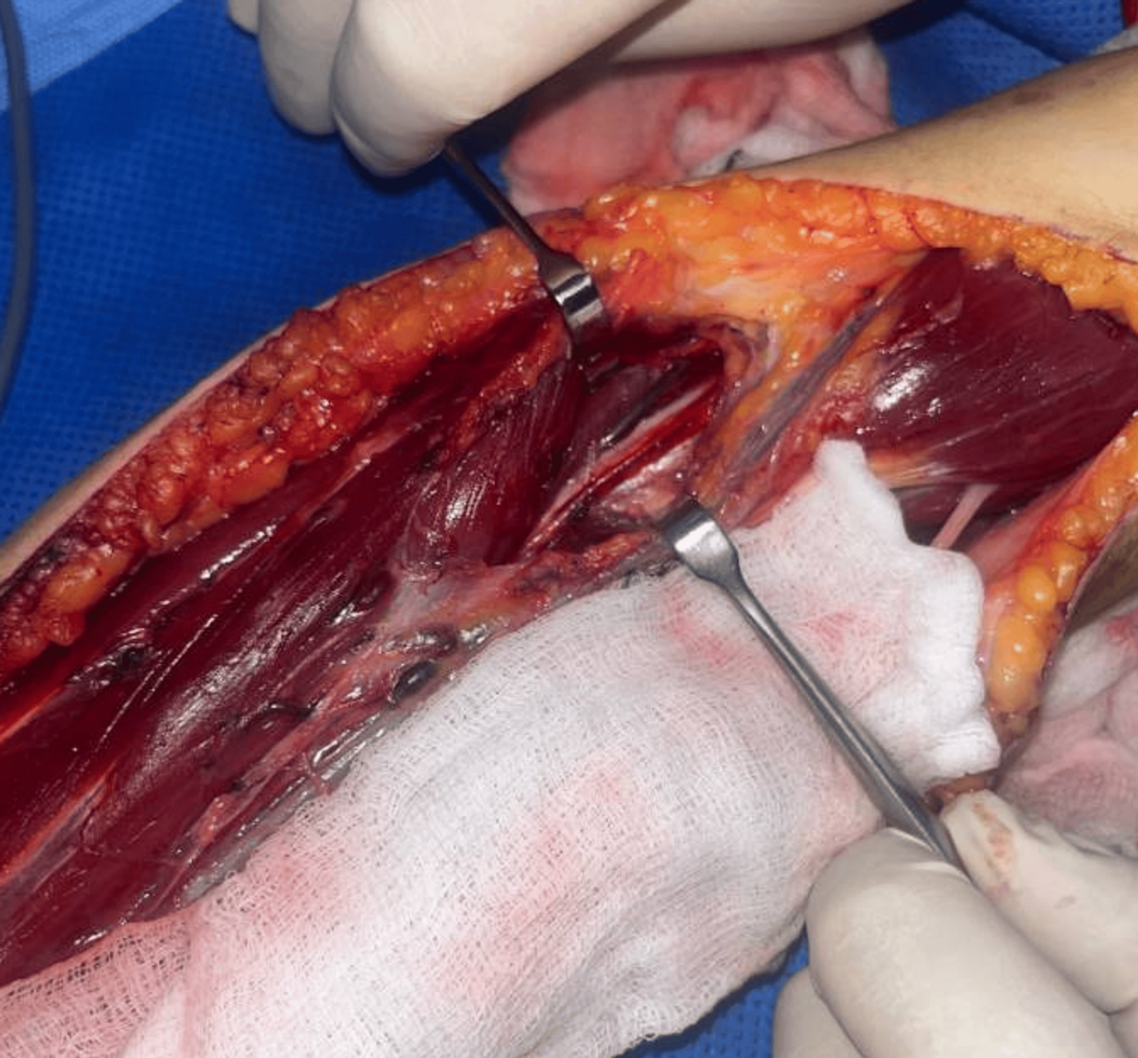
Intra-operative image showing the median nerve Exposure of the median nerve intra-operatively, some contusion was noted under magnified inspection.

The patient continued to experience severe pain in the left forearm and hand that required continuous local anaesthetic infusion via a brachial plexus catheter combined with intravenous patient-controlled oxycodone boluses and a ketamine infusion. Two days later, surgeons returned the patient to the theatre for fasciotomy closure. Intraoperative findings again demonstrated focal contusion and hyperaemia of the SBRN distal to the level of the radial head (Figures 3, 4) as well as an area of contusion of the median nerve (Figure 5). A cannula was inserted from the point of the electrode probe entry at the skin surface to a depth of 3 cm, the known length of the electrode probe needles, which aligned with the identified area of SBRN contusion. The compartments were closed easily, with no evidence of muscle swelling. At this stage, it was confirmed that this condition was not caused by compartment syndrome; therefore, an alternative pathology was investigated.

Investigations

Nerve conduction studies carried out 14 days after initial presentation showed limited, partial axonotmesis of the median nerve, particularly affecting the fascicles to the flexor pollicis longus and abductor pollicis brevis, with the assessment that there was an excellent prognosis for recovery. There was a possible lesion involving the radial nerve, which was felt to be of limited significance, and no abnormality of the ulnar nerve was found. Twenty-one days after the initial emergency department presentation, the patient’s pain was well controlled on oral analgesia. She was discharged with a comprehensive follow-up plan with the pain specialist team to monitor recovery over time, and will undergo serial nerve conduction studies.

## Discussion

This case demonstrates a rare but serious complication of BEST: median nerve axonotmesis, likely caused by unintended, irreversible electroporation from probe distortion. Although nerve injuries have been reported following other pulsed-field applications (e.g., electrochemotherapy, irreversible electroporation in oncology), they remain rare when proper electrode geometry is maintained [4,5]. A literature search of the PubMed repository database identified 20 articles using the search term “bleomycin electrosclerotherapy,” covering any articles published regardless of date. Nineteen of these articles describe treatment outcomes, and only one paper describes significant pain as a complication of treatment [3]; however, these cases were all managed with oral analgesia. No reported cases of pain severe enough to require hospital admission or surgical intervention were identified.

The case was discussed with the surgical team and the consultant who had performed the initial BEST, at which time multiple differential causes of the neuropathy were considered. Bleomycin-mediated tissue damage was felt unlikely, as bleomycin is only a weak sclerosant and did not fit the operative findings of a focally contused nerve. Compression within a fibrotic neocompartment was unlikely given that the external neurolysis did not improve symptoms. Direct mechanical needle trauma was ruled out based on operative findings (i.e., intact epineurium). In the case described in this article, the senior author concluded that there was no compartment syndrome, given the absence of elevated compartment pressures, no intraoperative evidence of tense compartments, and no relief of symptoms after fasciotomy. In conclusion, bleomycin toxicity, compartment syndrome, direct needle injury, and fibrotic compression were excluded based on the operative findings of a focal nerve contusion and lack of relief from comprehensive nerve decompression.

**Figure 6:**
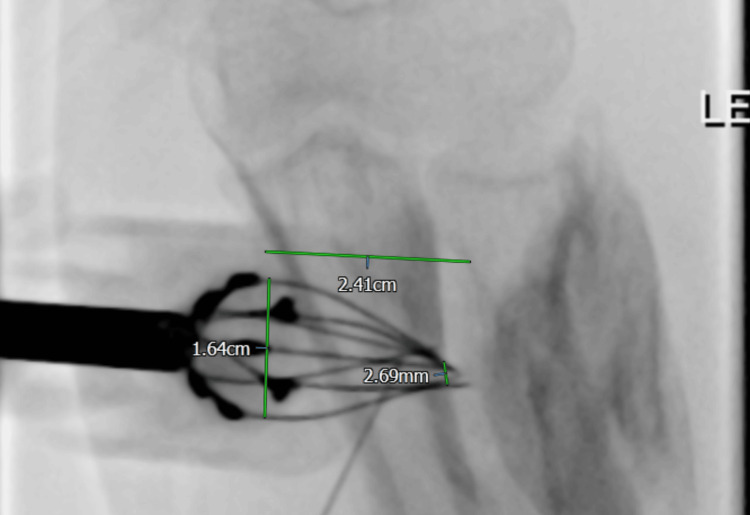
Fluoroscopic image of the electrostimulation probe's position Fluoroscopic image of the position of the electrostimulation probe demonstrating convergence of the electrodes close to the median nerve seen operatively and annotated with measurements.

The fluoroscopic images from the BEST treatment were obtained, from which a significant convergence of the needles of the hexagonal probe (Figure 6) was noted. Reliable electric field strength generation is based on a fixed probe geometry, with field strength increasing proportionally to a reduction in electrode distance. It is proposed that this change in probe geometry created an increase in the delivered electric field strength, which may have exceeded the threshold required to cause irreversible electroporation. This would then have resulted in a focal, athermal electrical injury at the probe tip, resulting in irreversible electroporation, causing an axonal injury. This pathophysiological course of events would explain the contused nerve segment and subsequent axonotmesis as identified during nerve conduction studies.

Given that a uniform electrical field strength between electrodes is calculated as E = V/d, where E is the field strength (V/m^2^), V is the voltage difference between electrodes, and d is the distance between the electrodes, any reduction in distance between the electrodes results in a proportional increase in field strength, given a constant voltage. The parameters that induce irreversible electroporation depend on tissue characteristics and the number, duration, and field strength of the delivered pulses. Some studies aiming to achieve irreversible electroporation have achieved it at field strengths as low as 820 V/cm [6], so a significant reduction in the distance between the probe electrodes could have the effect of unintended irreversible electroporation. BEST protocols typically apply up to 1,000 V/cm for reversible electroporation [7], leaving a potentially narrow therapeutic range. Even a modest deformation of probe geometry can lead to an increase in local field strength into the irreversible electroporation range. This highlights the safety-critical nature of maintaining probe geometry.

The pre-set geometry of the hexagonal electrode places the needles farther apart than the linear electrode series, so higher voltages are needed to achieve electroporation. The Igea Cliniporator measures electric current in real time during pulse delivery and is designed to cut out if thresholds for irreversible electroporation are exceeded. However, with a narrow therapeutic range, it is conceivable these thresholds could be crossed. Possible precautions to minimise the risk of nerve injury include the following: (1) Avoid higher voltage fixed geometry hexagonal electrodes when treating lesions close to named nerves, (2) If using fixed geometry electrodes near nerves, the maintenance of probe geometry must be confirmed on imaging prior to delivery of current to ensure a safe electric field strength is generated, (3) When treating lesions in contact with nerves, consider using single-needle electroporation under ultrasound guidance to ensure that the nerve remains at the periphery of the cliniporation zone.

## Conclusions

BEST is an effective treatment for lymphovascular malformations and is derived from the established electrochemotherapy technique. It demonstrates a low complication rate with only minor complications described in the literature. Regardless, this case highlights that there is a risk of a more significant complication in the form of athermal nerve injury with this treatment modality.

In conclusion, the surgical and radiographic evidence, as well as the timeline involved, support a diagnosis of athermal electrical injury causing median nerve axonotmesis. The most plausible explanation is that this occurred as a result of a geometric deformation of the treatment probe, amplifying the electric field beyond the therapeutic range, resulting in an unintended irreversible electroporation. Given that this is not a described complication of BEST treatment, it is hoped that this case study will highlight this risk and allow for discussion of this possibility during the consent process for this procedure. Therefore, clinicians must be aware that severe post-BEST neuropathic pain, in the absence of elevated compartment pressures, should lead to the suspicion of a direct nerve injury and should be promptly evaluated with neurological assessment or surgical exploration.
